# Association of Cardiac and Pulmonary CT Imaging Features with Respiratory Side Effects After Whole-Breast Radiotherapy

**DOI:** 10.3390/cancers18111727

**Published:** 2026-05-25

**Authors:** Marco Fois, Alfonso Belardo, Andrei Fodor, Lucia Perna, Laura Giannini, Paola Mangili, Gabriele Palazzo, Marcella Pasetti, Miriam Torrisi, Roberta Tummineri, Maria Giulia Ubeira-Gabellini, Antonella Del Vecchio, Nadia Gisella Di Muzio, Tiziana Rancati, Claudio Fiorino

**Affiliations:** 1 Medical Physics, IRCCS San Raffaele Scientific Institute, Via Olgettina 60, 20132 Milan, Italy; fois.marco@hsr.it (M.F.);; 2 Radiation Oncology, IRCCS San Raffaele Scientific Institute, 20132 Milan, Italy; 3 Faculty of Medicine and Surgery, Vita-Salute San Raffaele University, 20132 Milan, Italy; 4 Data Science Unit, Fondazione IRCCS Istituto Nazionale dei Tumori, 20133 Milan, Italy

**Keywords:** breast cancer, radiotherapy, lung side effects, cardiac calcifications, predictive modelling, hypofractionation

## Abstract

**Purpose:** This paper aimed to identify dosimetric, clinical, and CT-based densitometric predictors of radiation-induced pulmonary events in breast cancer patients treated with moderately hypofractionated radiotherapy. **Materials and Methods:** A single-institution cohort of 1172 consecutive patients treated with 3D conformal whole-breast radiotherapy (40 Gy/15 fractions) before 2017 was analyzed. Ipsilateral lung DVHs and CT densitometry metrics were extracted. Clinical variables and cardiac calcification (CAC) scores (Agatston_score, CAC_volume, Max_HU_Heart) were included. Univariable and multivariable logistic regressions were performed; collinearity was assessed via Spearman correlation and VIF. Optimal thresholds were derived using the Youden index. Internal validation used bootstrap resampling. **Results:** After a median follow-up of 6.5 years, 18 patients developed moderate/severe pulmonary events. The univariable analysis showed associations with lung densitometric features (median/mean HU, 10th percentile, the lung volume with HU < −850 (V850)), V37 Gy, lung volume, and CAC scores. Lower lung HU values and larger lung volumes were linked to higher risk. The best models combined V850 (or lung volume) with a CAC metric. The model including V850 > 175 cc and continuous Max_HU_Heart achieved an optimism-corrected AUC of 0.68, with good fit and calibration (Hosmer–Lemeshow *p* = 0.33, R^2^ = 0.847). **Conclusions:** The baseline cardiopulmonary status, captured by lung and heart densitometry, predicts pulmonary toxicity better than dosimetry. V850 > 175 cc was associated with a 4-fold higher risk, consistent with air trapping, known as a marker of emphysema.

## Simple Summary

In a single-institute cohort of 1172 patients receiving moderately hypofractionated radiotherapy to the whole breast (WBRT), pulmonary side effects were uncommon (1.5% of the total) but clinically relevant. Lung and heart densitometry features extracted from planning CTs, such as the low-density lung volume (HU < −850) and cardiac calcification scores, resulted in strong predictors of toxicity rather than radiation dose parameters. These results suggest that, in modern cohorts, most of the respiratory events after WBRT seem to be mainly related to baseline clinical conditions captured by CT imaging instead of being caused by radiation.

## 1. Introduction

Breast cancer is the most diagnosed malignancy among women worldwide and remains a leading cause of cancer-related mortality. Radiotherapy (RT) represents a cornerstone of breast-conserving treatment, significantly reducing the risk of local recurrence and improving long-term survival [1,2]. Modern RT techniques have markedly improved in terms of dose conformity and normal tissue sparing; however, treatment is still associated with a spectrum of adverse effects. Acute and subacute toxicities prevalently involve the skin and the irradiated breast, manifesting as erythema, desquamation, edema, and pain. Beyond these transient toxicities, increasing attention has been directed toward more severe, potentially long-term complications involving the heart and lungs. Incidental irradiation of the heart has been linked to an increased risk of ischemic heart disease, valvular dysfunction, and other cardiovascular sequelae [3,4]. Similarly, the lung parenchyma, partially included within the tangential breast fields, can be exposed to clinically relevant radiation doses, predisposing patients to radiation-induced pneumonitis and, in some cases, to persistent fibrosis [5]. These pulmonary toxicities can significantly impact quality of life and may limit future therapeutic options, underscoring the clinical relevance of improving risk stratification [2,6,7]. In this context, the relationship between lung dose-volume metrics and radiation-related pulmonary side effects has been extensively investigated, mostly in studies involving lung cancer patients [8,9,10,11]. Among the considered dosimetry variables, the mean lung dose (MLD) and the portion of lung volume receiving more than the given dose levels (VxGy), are the most analyzed [8,9,10]. Despite several studies reporting dose–response relationships involving MLD and V20 Gy, no accepted dose-volume thresholds have been yet universally established, with substantial differences across the literature [11].

This variability can be associated with differences in treatments, fractionation schemes, dose-calculation methods or even diversity in side effect scoring systems. Moreover, both DVH-based parameters and more advanced modeling techniques have shown partial ability to fully capture individual susceptibility, suggesting that dose-volume information alone may be insufficient to reliably predict lung side effects, especially in clinical settings characterized by relatively low lung-dose, as in the adjuvant breast RT context [11,12,13,14].

The scenario regarding pulmonary toxicity after whole breast RT (WBRT) is very different compared to lung or other thoracic malignancies: the “regular” position and shape of PTV translates into the irradiation of relatively small portions of the ipsilateral lung, with similar spatial distribution of the high-dose region. As stated, the radiological evidence of pneumonia and fibrosis has been largely studied [15] and mostly dealt with sub-clinical or minimally impacting effects in terms of quality-of-life.

On the other hand, studies dealing with clinically relevant respiratory symptoms are scarce [16], also because of the expected relatively rare occurrence after adjuvant WBRT. Then, identifying reliable features associated with lung side effects after breast radiotherapy remains a major challenge. Traditional dosimetric parameters may provide only a partial picture, as individual susceptibility may in principle largely modulate the risk; this suggests that biological or imaging-based markers may be regarded to complement dose metrics in predicting adverse outcomes. Quantitative lung densitometry extracted from CT imaging and metrics describing cardiac calcifications have recently emerged as promising candidates, potentially capturing pre-existing subclinical vulnerability that interacts with radiation dose distributions [17,18]. The aim of this work is to investigate dosimetric, densitometric, and imaging parameters extracted from planning CT as potential predictors of moderate/severe respiratory events in patients treated with adjuvant WBRT.

## 2. Materials and Methods

### 2.1. Cohort Information

All data were extracted from a prospectively maintained institutional database including all breast cancer patients treated with postoperative radiotherapy since 2009. For the present analysis, we selected women who received hypofractionated WBRT (40 Gy in 15 fractions) without regional nodal irradiation. Patients who underwent a boost to the tumor bed were also excluded. We considered only patients treated with 3DCRT tangential fields in the period 2009–2017, owing to an Institutional Board approved retrospective study (ClinicalTrials.gov: NCT03077191) and previously analyzed concerning skin/breast toxicities and cardiac events [19,20,21,22,23,24]. A total of 1200 patients met the initial criteria; 28 were excluded due to bilateral radiotherapy (synchronous or sequential), resulting in a final cohort of 1172 women (right-sided: 569; left-sided: 603).

Details on contouring, treatment planning and delivery have been previously described [19,20,21,22]. In short, all patients underwent CT simulation on the same scanner (Light Speed GE MDCT, GE HealthCare Technologies Inc., headquarters in Chicago, IL, USA) and were planned with manually optimized 3D-CRT using tangential fields. The clinical target volume (CTV) encompassed the whole breast according to national guidelines [25]. A planning target volume (PTV) was then generated by expanding the CTV by 5–15 mm, except posteriorly toward the lung (5 mm), and cropped to exclude the outer 5 mm of skin. Planners typically used a median of four segments to achieve homogeneous dose coverage (V95% > 95%, hot spots < 108%). Treatment delivery was performed using a Varian DHX 6-MV linac, (Varian Medical Systems Inc., headquarters in Palo Alto, CA, USA), with daily CBCT-based image guidance. Planning data, made with Eclipse TPS v13.7 were retrieved, and 3D dose distributions were imported into MIM software (v7.2.8).

Major patient characteristics, including pulmonary dosimetry, densitometry and clinical factors, are summarized in Table 1. Smoking status was also considered in detail: only actively smoking individuals at the time of anamnesis were defined as smokers, and for the present analysis, active and former smokers were pooled. Comorbidities such as obesity, diabetes and hypertension were all self-reported during anamnesis and included if previously diagnosed.

### 2.2. Recovery of Pulmonary Side Effects

Pulmonary events were collected following the same procedural framework adopted for cardiac toxicity surveillance described in detail [20]. Specifically, radiation pneumonitis/pulmonary fibrosis was registered at each scheduled follow-up visit (6, 18, 30, 42, 54 and 66 months). In addition, respiratory events were also registered as the presence of ≥Grade 2 clinical respiratory symptoms and/or when pneumonia/fibrosis was observed on CT/X-ray performed for other reasons, according to Common Terminology Criteria for Adverse Events (CTCAE) score v.4.0, updated to v. 5.0 for all patients during the institutional reviews conducted in 2019 and subsequently in 2023.

### 2.3. Dosimetry and Densitometry Information

For all patients, we extracted dosimetry and densitometry data for lungs using in-house Python, (v3.11) scripts for both lungs (ipsi- and contralateral) and for paired lungs. In particular, these features included the total lung volume (Volume_Lung), the fractions of lung volume in cc and % receiving selected dose levels (from V10 Gy to V40 Gy), the mean lung dose (MLD) and key pulmonary statistical densitometry parameters expressed in Hounsfield Units (HU), such as the Max_HU, Mean_HU, Median_HU, as well as the percentiles p10%_HU, p25%_HU, p75%_HU and p90%_HU. In addition, the HU histograms of ipsilateral lungs of patients with and without toxicity were compared by the Mann–Witney test. The HU value corresponding to the minimum *p*-value of the test was also included as a potential predictor (resulting in HU = −850; V850). Both the dosimetry and densitometry variables are summarized in Table 1 and, along with cardiac parameters, were considered as features potentially associated with respiratory side effects [25,26,27,28,29,30,31,32,33]. CAC were identified by using an in-house Python script which classified calcified lesions as pixels with HU > 130 and area ≥ 1 mm^2^ or ≥4 adjacent pixels, according to [26,27]. The script evaluated all calcium deposits within the heart, automatically segmented using commercial software (MIM_Protégé, MIMv7.2.8), including those located in the coronary arteries, valves, and myocardium; key metrics such as the Agatston_score (AS), CAC volume, and CAC maximum HU (Max_HU_Heart) were then assessed [33].

### 2.4. Statistical Modeling and Analyses

The following variables were considered: Max_HU_Heart, Agatston_score, Volume_Lung, Lung Mean_HU, Lung_Median_HU, V850, p10%_HU, p25%_HU, p75%_HU and p90%_HU, the (absolute and %) fraction of volumes from V10 Gy to V40 Gy, smoking status, hypertension, diabetes, chemotherapy, hormone therapy and obesity. All considered variables were then tested in univariable logistic regression, adopting a significance threshold of *p* < 0.05. For multivariable modelling, only predictors with an univariable significance level below 0.10 were considered. Stepwise multivariable logistic regression analyses were performed using MedCalc (v22) and, to mitigate any risk of overfitting, a maximum value of two variables was considered. Before the stepwise selection, collinearity among features was assessed using the Variance Inflation Factor (VIF) and pairwise Spearman correlation coefficients: variable pairs showing evidence of multicollinearity were excluded, defined as either an absolute Spearman correlation coefficient > 0.75 or a VIF exceeding 2. In absence of multicollinearity, both densitometric metrics and CAC scores were included in different bivariable models and the best-performing couples, in terms of model discrimination, were assessed using the ROC-AUC [34,35,36]. To confirm the stability, a bootstrap resampling procedure with 1000 iterations was applied, as internal validation according to TRIPOD guidelines [37], yielding optimism-corrected AUC [38,39,40,41]. This procedure was chosen due to the low number of events and performed with an in-house code (MedicalAI—Medical Artificial Intelligence Toolkit for Research, http://github.com/pymaitre [21], accessed on 20 May 2026) with model coefficients estimated through maximum likelihood. Calibration was assessed to verify the agreement between predicted and observed events. Variables showing the strongest and most consistent statistical significance were further dichotomized using the Youden index to identify optimal cut-off values.

## 3. Results

The median follow-up was 6.5 years (IQR 5.9–8.8). In total, 18/1172 (1.5%) ≥ G2 respiratory events were identified. These events included, among others, radiation pneumonitis, pleuritis, acute exacerbations of chronic obstructive pulmonary disease (COPD), pulmonary edema, and recurrent bronchitis, as summarized in Table 2.

As shown in Table 2, only a minority of the documented respiratory events can be considered unequivocally as caused by Radiotherapy. Many events may instead reflect post-RT worsening or exacerbation of pre-existing clinical conditions.

**Figure 1:**
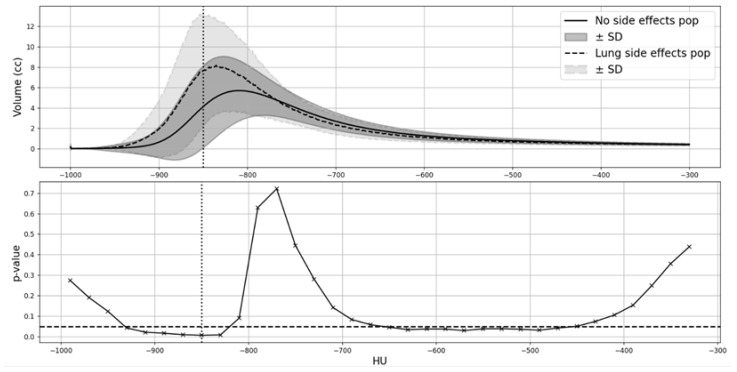
On the top panel, HU distributions for the population with and without side effects (mean value and CI). On the bottom panel, the corresponding *p*-value trend is shown with a cross-marked line.

**Figure 2:**
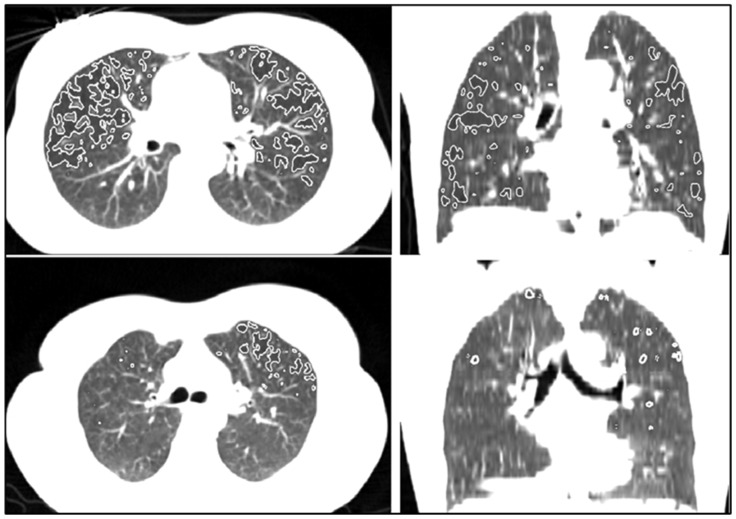
V850 region segmentation on CT scans of two representative patients of the same age for HU ranging from −1000 to −500. On the top panel a patient with pulmonary side effects (V850 = 177 cc), on the bottom panel a patient with no recorded side effects (V850 = 57 cc).

Concerning densitometry lung information, we focused on the ipsilateral lung; the same analyses were repeated for the contralateral lung. In addition, we have also considered both lungs as a unique structure. In the analysis of the combined lungs, dosimetric variables were not considered as their impact on the contralateral lung is negligible. Results regarding contralateral and both lungs’ analyses are shown in Section S1 of the Supplementary Materials. The HU histogram differences between patients with and without events showed a clearly different shape. The most significant and large differences were seen in the range −920 < HU < −820, with the minimum *p*-value = 0.0052 for HU = −850, as shown in Figure 1). As an explicative example, Figure 2 shows two different patients’ CTs (with and without pulmonary side effects) with “high” and “low” V850, respectively 177 cc and 57 cc, selecting the same HU window (−1000, −500) to enhance the different densitometry patterns. The results of the univariable and bivariable logistic regression analyses are summarized in Table 3 and Table 4, respectively. At univariable analysis, lung volume and CAC scores demonstrated the strongest associations in terms of odds ratios, significance, and discrimination capability. Among the other available variables, V37 Gy (cc) was of borderline significance while MLD > 5.5 Gy, hypertension and smoking showed a trend (*p*-value = 0.17 and 0.20 respectively) with ORs around 2. Specifically, lung volume showed an AUC of 0.68 (*p*-value = 0.008), while the Max_HU_Heart from CAC scoring yielded an AUC of 0.64 (*p*-value = 0.004). The Agatston_score also demonstrated good discrimination (AUC = 0.69, *p*-value = 0.001). In addition, the Lung Mean_HU value was significantly associated with pulmonary toxicity (*p*-value = 0.018, OR = 0.987, AUC = 0.67). A summary of the univariable analysis carried out on all the VxGy variables, expressed in cc and %, is shown in Section S1 of the Supplementary Materials.

The optimal cut-off values were: 1745 cc for lung volume, 175 cc for V850, 232 HU for Max_HU_Heart, 7.63 for the Agatston_score, 5.5 Gy for the MLD and 114.6 cc, 111.5 cc and 50.8 cc for the V36, V37 and V38 Gy, respectively. Patients with lung volume > 1745 cc had a markedly increased risk of pulmonary toxicity (*p*-value = 0.0003, OR = 6.21, AUC = 0.69). Max_HU_Heart > 232 was associated with a moderate risk (*p*-value = 0.004, OR = 2.64, AUC = 0.61), whereas Agatston_score > 7.63 showed much higher OR (*p*-value = 0.0005, OR = 6.54, AUC = 0.70). Similarly, Lung Mean_HU values less than −720.9 HU showed strongly increased risk of side effects (*p*-value = 0.0005, OR = 5.55, AUC = 0.69).

Lung Mean_HU and Volume_Lung were identified as collinear, exhibiting a strong negative correlation, and were therefore not jointly included in bivariable models. Despite the fact that the smoking status was not retained in the two variables model due to a *p*-value = 0.2, its correlation with Volume_Lung and Mean_HU was tested. Spearman analysis showed no relevant association between smoking and Mean_HU (ρ = −0.012) and Volume_Lung (ρ = 0.117), even if smokers presented a significant difference (*p*-value = 0.0001) in lung volume compared to non-smokers, with a median of 1440 cc and 1329 cc, respectively. The variables diabetes, obesity, hypertension and age were also initially considered in the multivariable model but were excluded by the stepwise analysis.

**Figure 3:**
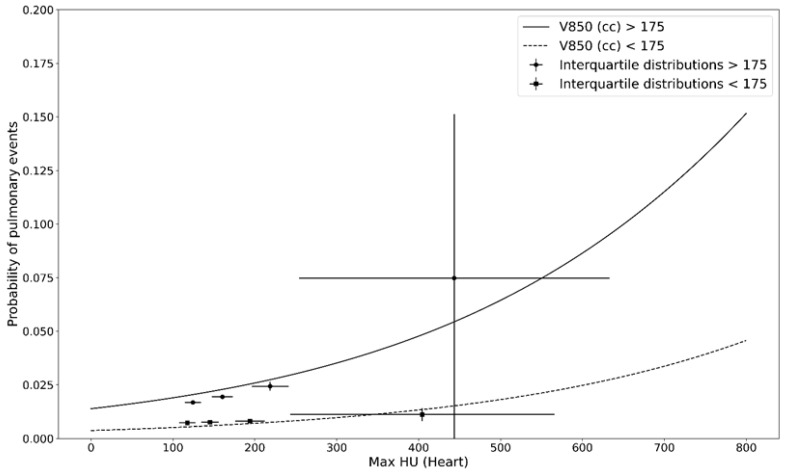
Risk of late pulmonary events: cardiac Max_HU_Heart stratified by lung volume V850 above or below 175 cc. The curves represent model-predicted probabilities, while the points indicate observed event incidence across cohort quartiles with associated 95%CI.

Several combinations involving cardiac calcification metrics and lung densitometry variables yielded highly significant models, while V37 Gy was never retained among the two best discriminative features. Among the different bivariable models considered, only two were selected: the first combining V850 > 175 cc and Agatston_score and the second combining V850 > 175 cc and Max_HU_Heart. To favor clinical interpretability, we selected the second model as a compromise between statistical performance and ease of communication. The performance of the selected model is reported in Table 4, while the corresponding calibration plot is shown in Section S3 of the Supplementary Materials. Figure 3 displays the predicted probability curves for the proposed model, constructed by sampling events above and below the best cut-off value for V850 (175 cc).

The performance of the alternative model considering Agatston_score instead of Max_HU_Heart, which shows an optimism-corrected AUC of 0.72, is described in Section S2 of the Supplementary Materials. In this context, it is also important to point out that, despite the fact that the observed AUC was moderate, this result should be interpreted considering the very low event rate of the analyzed cohort (1.5% of the total population). In settings involving rare side effects, model calibration may represent a particularly relevant aspect, and the good agreement achieved between predicted and actual risks suggests a potentially useful role for preliminary risk stratification, while further validation in larger independent cohorts remains mandatory.

Similar analyses were also conducted on the combined lung structure, in which the cut-offs for the two variables were identified using the Youden index (V850 > 291.7 cc). As expected, similar results were obtained: for the model applied to the combined lung structure, we observed a *p*-value = 0.0004 and an apparent AUC = 0.71.

## 4. Discussion

Despite the reported variability in assessing pulmonary side effects after breast RT, the low rate of events in our population (i.e., 1.5%) is consistent, based on NTCP models [42,43], with the “low” MLD values of our modern cohort (mean: 5.4 Gy). However, to place these results into a broader clinical framework, it is important to contextualize the pulmonary events observed in our cohort, by considering the baseline incidence of similar conditions in the age-matched general population. Epidemiological studies show that the incidence of asthma-COPD overlaps in the general population is estimated to be approximately 0.6–2.1 cases per 1000 person/year [44], while idiopathic pulmonary fibrosis is even rarer, with an incidence of 0.22–7.4 cases per 100,000 person/year [45]. These data seem to suggest that the background incidence of most non-radiation related pulmonary side effects is relatively low, supporting the interpretation that the overall pulmonary event rate observed after breast RT only slightly exceeds what would be expected based solely on the general background risk, although causality cannot be inferred for individual non-specific events [46].

Outside their direct impact on respiratory function, radiation-induced lung injury and/or fibrosis may also contribute to the development of a pro-tumorigenic microenvironment via chronic inflammation or macrophage activation. Moreover, fibrotic tissue remodeling also appears to have a key role in this sense [47]. Experimental evidence suggests that these processes could potentially facilitate the conditions for tumor recurrence and metastatic spread, further supporting the clinical relevance of stratifying and isolating patients at higher risk of respiratory side effects after RT [47].

Looking at potential lung dosimetry predictors, intriguingly, and not unexpectedly, the most significant features are V37 Gy and V38 Gy (in cc), roughly corresponding to the lung volume receiving a dose value near to the prescribed dose. This result is consistent with the well assessed relationship between the fraction of lung volume included in the tangential fields and the risk of pneumonia and fibrosis [48,49]. On the other hand, despite the fact that their OR are relatively high (1.008 per cc), V37 Gy and V38 Gy were not retained in bivariable analyses, showing that non-dosimetry factors related to the baseline respiratory and cardiac conditions are stronger. The lack of any association between %DVH and respiratory events corroborates this statement. In fact, compared to older studies [49], the current fraction of lung volume included in the high-dose regions has been dramatically reduced, and our results of a “weak” association is consistent with it, even if it is important to note that these results can be attributed to the very low heterogeneity of the variables in our cohort, as observed for MLD. This phenomenon could negatively affect and damp the response of dosimetry features. These findings may also have practical implications for treatment personalization. While it is unclear if it may help in the selection of fractionation schedules, the identification of patients at higher risk based on CT biomarkers may ideally help in selecting the best treatment technique, in identifying best candidates for DIBH and in the choice to re-irradiate patients with secondary sarcomas [50]. On the other hand, it is important to underline that the interplay between the presence of the CT-based negative predictors and the dose to the lung could not be assessed in the current cohort.

Our analysis identified clear association between respiratory side effects and several imaging biomarkers, including lung volume, CAC metrics (Max_HU_Heart and Agatston_score) and lung densitometry features. Interestingly, a negative association between lung volume and risk was found, while a larger volume of low-density lung (V850) was a highly significant risk factor. These findings support the idea that lung densitometry may reflect underlying sub-clinical baseline respiratory problems. Indeed, some pathological conditions, such as emphysema, are known to be associated with lung HU density histogram shifted toward lower values and increased lung volume [51,52,53]. Structural damage to the alveolar walls leads to their weakening and possible rupture, resulting in the formation of larger air spaces, contributing to the phenomenon referred to as hyperinflation [51]. The enlarged air spaces promote air trapping, which hinders the entry of fresh, oxygen-rich air into the affected regions of the lungs. Consequently, the effective surface area available for gas exchange can be significantly reduced, impairing the oxygenation of the blood. From a clinical point of view, early pathophysiological changes captured by CT may predict the likelihood of experiencing dyspnea and other respiratory problems. The air trapping effect, well described in other contexts using CT taken at deep inspiration for emphysema quantification and CT at expiration for air trapping assessment, is consistent with our findings and offers a quite convincing clinical explanation. Consistent with our results, CT scans taken during free breathing, such as our planning scans, and expiratory CT scans were reported to yield almost identical results in the diagnosis of air trapping in emphysema [52].

The predictive role of cardiac calcification metrics was especially notable. While CAC scoring has been reported to be associated with the risk of cardiovascular disease after breast cancer RT [19,32,33,41], the current finding, reported here, to our knowledge, for the first time, is not surprising and confirms the hypothesis that pre-existing sub-clinical cardiovascular problems, reflected by the presence of calcifications, are also potentially linked to an increased risk of pulmonary events.

Regarding the proposed bivariable models, they demonstrated good discrimination capacity and excellent calibration. Their robust performance provides preliminary support for integrating cardiac and pulmonary imaging biomarkers into predictive frameworks for radiation-induced respiratory toxicity. However, due to the exploratory nature of this work and the limited number of events, the proposed predictive models should be validated in independent and larger cohorts.

## 5. Conclusions

According to our analyses, combining lung- and heart-related CT biomarkers enhances the prediction of the risk of experiencing respiratory symptoms after breast cancer RT. The suggested bivariable model showed good discrimination and excellent calibration, confirming potential clinical relevance. Future research could investigate mechanistic pathways and assess how these biomarkers could support more personalized radiotherapy planning and post-treatment surveillance. In any case, studies involving larger populations could also clarify interconnections with lung dosimetry parameters, since the absolute volumes of lung included in the high-dose region (V37 Gy and V38 Gy) were associated with a three-time risk of experiencing respiratory problems, although their importance was outperformed by the imaging-based predictors. Similar considerations may be valid for the impact of smoking.

This work is part of the EU-funded TETRIS project [54], merging data with other large cohorts, totaling over 4000 patients. Within this framework, future analyses will further investigate the interplay between pulmonary damage and effects on cardiac structures and sub-structures, further elucidating cardiopulmonary risks associated with breast radiotherapy.

## Figures and Tables

**Table 1:** Clinical, dosimetric and densitometric characteristics of the cohort (*n* = 1172).

Clinical Features	
**Age groups (yrs)**	
Mean	60.8
Median [IQR]	62 [51;70]
<45	118 (10.1%)
45–55	273 (23.3%)
55–65	285 (24.3%)
>65	496 (42.3%)
**Lesion position**	
Left	603 (51.5%)
Right	569 (48.5%)
**Chemotherapy**	
Anthracycline	220 (18.8%)
Concurrent Chemo/RT	29 (2.4%)
**Other therapies**	
Trastuzumab	100 (8.5%)
Hormonal	939 (80.1%)
**Health conditions**	
Smoking	237 (20.2%)
Diabetes	166 (14.2%)
Obesity	272 (23.2%)
Hypertension	404 (34.4%)
**Dosimetric/Densitometric features**	
**Mean Heart Dose (MHD)**	
Mean (SD) [Gy]	1.72 (1.23)
MHD > 1 Gy	640/1172 (54.6%)
**Mean Ipsilateral Lung Dose (MLD)**	
Mean (SD) [Gy]	5.42 (1.64)
MLD > 5 Gy	714/1172 (60.9%)
**Mean Ipsilateral Irradiated Volume (cc)**	
V10 Gy (SD)	193.88 (83.56)
V15 Gy (SD)	173.29 (77.62)
V20 Gy (SD)	154.92 (73.44)
V25 Gy (SD)	139.11 (69.39)
V30 Gy (SD)	123.56 (65.23)
V35 Gy (SD)	100.74 (58.75)
V37 Gy (92.5% target dose) (SD)	80.58 (53.21)
V38 Gy (95% target dose) (SD)	62.74 (48.85)
V39 Gy (97.5% target dose) (SD)	39.31 (40.67)
V40 Gy (SD)	15.83 (25.19)
**Mean Pulmonary Densitometry**	
**Ipsilateral Lung**	
Volume lung (cc) (SD)	1390.30 (345.07)
Max HU (SD)	739.65 (195.56)
Mean HU (SD)	−678.37 (47.83)
Median HU (SD)	−746.92 (51.08)
V850 (cc) (SD)	142.01 (202.53)
p10% HU (SD)	−831.07 (34.50)
p25% HU (SD)	−803.55 (39.60)
p75% HU (SD)	−627.37 (73.23)
p90% HU (SD)	−402.54 (65.43)
**Contralateral Lung**	
Volume lung (cc) (SD)	1369.35 (375.23)
Max HU (SD)	732.27 (207.52)
Mean HU (SD)	−678.45 (47.48)
Median HU (SD)	−746.94 (50.81)
V850 (cc) (SD)	140.24 (198.43)
p10% HU (SD)	−830.77 (34.71)
p25% HU (SD)	−801.10 (39.73)
p75% HU (SD)	−627.98 (72.23)
p90% HU (SD)	−402.84 (64.14)

**Table 2:** Summary of registered pulmonary events.

Type	*n*
Radiation pneumonitis	4
Non-radiation pneumonia, inflammation/infection episodes	4
Bronchitis/bronchiectasis	3
Pulmonary fibrosis	2
COPD/emphysema/asthma-related	1
Atelectasis	1
Pleuritis accompanied to pericarditis	1
Pulmonary embolism	1
Pulmonary edema	1

**Table 3:** Summary of the results of the univariable analysis. The odds ratio (OR) is expressed per cc for the Volume_Lung, and Mean_HU and per 1 HU for the cardiac Max_HU_Heart score. For the Agatston_score, the OR refers to the value calculated using the CAC area measured in mm^2^.

Continuous Variables	*p*-Value	OR	CI95%	AUC
Volume_Lung (cc)	0.008	1.0016	[1.001, 0.002]	0.68
V850 (cc)	0.02	1.002	[1.001, 1.003]	0.67
Lung Mean_HU	0.018	0.987	[0.976, 0.998]	0.67
p10%_HU	0.019	0.982	[0.967, 0.997]	0.66
Max_HU_Heart	0.004	1.003	[1.0015, 1.005]	0.64
Agatston_score	0.001	1.001	[1.0005, 0.002]	0.69
MLD (Gy)	0.87	1.025	[0.771, 1.362]	0.52
V36 Gy (cc)	0.059	1.007	[1.000, 1.014]	0.61
V37 Gy (cc)	0.050	1.008	[1.0003, 1.016]	0.61
V38 Gy (cc)	0.081	1.008	[0.999, 1.016]	0.61
V39 Gy (cc)	0.32	1.005	[0.995, 1.015]	0.58
**Dichotomous Variables**	***p*-Value**	**OR**	**CI95%**	**AUC**
Volume_Lung (cc) > 1745	0.0003	6.2	[2.4, 15.1]	0.69
V850 (cc) > 175	0.0047	4.1	[1.5, 10.7]	0.67
Lung Mean_HU < −720.9	0.0005	5.6	[2.2, 14.2]	0.69
p10%_HU < −846	0.032	2.8	[1.07, 7.2]	0.63
Max_HU_Heart > 232	0.004	2.6	[1.03, 6.6]	0.61
Agatston_score > 7.63	0.0005	6.5	[1.88, 22.7]	0.70
MLD (Gy) > 5.5	0.16	2.0	[0.74, 5.3]	0.58
V36 Gy (cc) > 114.6	0.042	2.7	[1.04, 6.8]	0.62
V37 Gy (cc) > 111.5	0.023	3.0	[1.19, 7.7]	0.63
V38 Gy (cc) > 50.8	0.033	3.1	[1.0, 9.3]	0.63
V39 Gy (cc) > 31.7	0.075	2.4	[0.89, 6.4]	0.61
**Clinical Variables**	***p*-Value**	**OR**	**CI95%**	**AUC**
Smoke	0.20	2.0	[0.74, 5.4]	0.57
Chemotherapy	0.57	0.7	[0.24, 2.2]	0.53
Hormone therapy	0.72	1.3	[0.36, 4.3]	0.52
Diabetes	0.35	1.8	[0.57, 5.4]	0.54
Hypertension	0.17	1.9	[0.76, 4.9]	0.58
Obesity	0.65	1.3	[0.45, 3.6]	0.52
Age	0.19	1.02	[0.98, 1.07]	0.57

**Table 4:** Summary of the bivariable logistic regression model. The results are reported using two rows to show the contribution of each variable. Here, var. *p*-value refers to the variable *p*-value, while mod. *p*-value is the regression model’s *p*-value. OR refers to the Odd Ratio of the model, β denotes the coefficients of the logistic regression, HL is the Hosmer–Lemeshow test, while O.C. AUC and app. AUC are the optimism-corrected AUC after 1000 bootstrap iterations and apparent AUC, respectively.

Variable	*p*-Value (Var)	*p*-Value (Model)	OR	CI95%	Coeff. β	*p*-Value (HL)	O.C. AUC (App.AUC)
**V850 > 175 cc** **Max_HU_Heart**	0.009 0.0015	0.0004	3.72 1.003	[1.39, 9.96] [1.001, 1.005]	1.314 0.0032	0.33	0.68 (0.70)

## Data Availability

The data presented in this study are available on reasonable request.

## Supplementary Materials

The following supporting information can be downloaded at: https://www.mdpi.com/article/10.3390/cancers18111727/s1. The Supplementary Materials include the complete univariable logistic regression analysis (Section S1—Tables S1–S4), the results of the bivariable model related to the Agatston_score (Section S2—Table S5) and the plot of the probability risk curves (Section S2—Figure S1). Finally, the calibration plot of the bivariable model descripted in Section 3 is illustrated in Section S3—Figure S2.

## References

[1] European Cancer Information System (ECIS) (2020). Breast Cancer Factsheet. https://ecis.jrc.ec.europa.eu/sites/default/files/2023-12/Breast_cancer_en-Dec_2020.pdf.

[2] Allemani C., Matsuda T., Di Carlo V., Harewood R., Matz M., Nikšić M., Bonaventure A., Valkov M., Johnson C.J., Estève J. (2018). Global surveillance of trends in cancer survival 2000–14 (CONCORD-3): Analysis of individual records for 37 513 025 patients diagnosed with one of 18 cancers from 322 population-based registries in 71 countries. Lancet.

[3] Darby S.C., Ewertz M., McGale P., Bennet A.M., Blom-Goldman U., Brønnum D., Correa C., Cutter D., Gagliardi G., Gigante B. (2013). Risk of Ischemic Heart Disease in Women after Radiotherapy for Breast Cancer. N. Engl. J. Med..

[4] Gagliardi G., Lax I., Ottolenghi A., Rutqvist L.E. (1996). Long-term cardiac mortality after radiotherapy of breast cancer—Application of the relative seriality model. Br. J. Radiol..

[5] Käsmann L., Dietrich A., Staab-Weijnitz C.A., Manapov F., Behr J., Rimner A., Jeremic B., Senan S., De Ruysscher D., Lauber K. (2020). Radiation-induced lung toxicity—Cellular and molecular mechanisms of pathogenesis, management, and literature review. Radiat. Oncol..

[6] Darby S., McGale P., Correa C., Taylor C., Arriagada R., Clarke M., Cutter D., Davies C., Ewertz M., Early Breast Cancer Trialists’ Collaborative Group (EBCTCG) (2011). Effect of radiotherapy after breast-conserving surgery on 10-year recurrence and 15-year breast cancer death: Meta-analysis of individual patient data for 10 801 women in 17 randomised trials. Lancet.

[7] Yee C., Wang K., Asthana R., Drost L., Lam H., Lee J., Vesprini D., Leung E., DeAngelis C., Chow E. (2018). Radiation-induced Skin Toxicity in Breast Cancer Patients: A Systematic Review of Randomized Trials. Clin. Breast Cancer.

[8] Tucker S.L., Li M., Xu T., Gomez D., Yuan X., Yu J., Liu Z., Yin M., Guan X., Wang L.-E. (2013). Incorporating Single-nucleotide Polymorphisms Into the Lyman Model to Improve Prediction of Radiation Pneumonitis. Int. J. Radiat. Oncol. Biol. Phys..

[9] Tucker S.L., Jin H., Wei X., Wang S., Martel M.K., Komaki R., Liu H.H., Mohan R., Chen Y., Cox J.D. (2010). Impact of Toxicity Grade and Scoring System on the Relationship Between Mean Lung Dose and Risk of Radiation Pneumonitis in a Large Cohort of Patients with Non–Small Cell Lung Cancer. Int. J. Radiat. Oncol. Biol. Phys..

[10] Stenmark M.H., Cai X.-W., Shedden K., Hayman J.A., Yuan S., Ritter T., Ten Haken R.K., Lawrence T.S., Kong F.-M.S. (2012). Combining physical and biologic parameters to predict radiation-induced lung toxicity in patients with non-small-cell lung cancer treated with definitive radiation therapy. Int. J. Radiat. Oncol. Biol. Phys..

[11] Zhao J., Yorke E.D., Li L., Kavanagh B.D., Li X.A., Das S., Miften M., Rimner A., Campbell J., Xue J. (2016). Simple Factors Associated with Radiation-Induced Lung Toxicity After Stereotactic Body Radiation Therapy of the Thorax: A Pooled Analysis of 88 Studies. Int. J. Radiat. Oncol. Biol. Phys..

[12] Borst G.R., Ishikawa M., Nijkamp J., Hauptmann M., Shirato H., Onimaru R., van den Heuvel M.M., Belderbos J., Lebesque J.V., Sonke J.-J. (2009). Radiation pneumonitis in patients treated for malignant pulmonary lesions with hypofractionated radiation therapy. Radiother. Oncol..

[13] Chang J.Y., Li Q.-Q., Xu Q.-Y., Allen P.K., Rebueno N., Gomez D.R., Balter P., Komaki R., Mehran R., Swisher S.G. (2014). Stereotactic Ablative Radiation Therapy for Centrally Located Early Stage or Isolated Parenchymal Recurrences of Non-Small Cell Lung Cancer: How to Fly in a “No Fly Zone”. Int. J. Radiat. Oncol. Biol. Phys..

[14] Guckenberger M., Baier K., Polat B., Richter A., Krieger T., Wilbert J., Mueller G., Flentje M. (2010). Dose–response relationship for radiation-induced pneumonitis after pulmonary stereotactic body radiotherapy. Radiother. Oncol..

[15] Wennberg B., Gagliardi G., Sundbom L., Svane G., Lind P. (2002). Early response of lung in breast cancer irradiation: Radiologic density changes measured by CT and symptomatic radiation pneumonitis. Int. J. Radiat. Oncol. Biol. Phys..

[16] Mangesius J., Minasch D., Fink K., Nevinny-Stickel M., Lukas P., Ganswindt U., Seppi T. (2023). Systematic risk analysis of radiation pneumonitis in breast cancer: Role of cotreatment with chemo-, endocrine, and targeted therapy. Strahlenther. Onkol..

[17] Mukesh M.B., Harris E., Collette S., Coles C.E., Bartelink H., Wilkinson J., Evans P.M., Graham P., Haviland J., Poortmans P. (2013). Normal tissue complication probability (NTCP) parameters for breast fibrosis: Pooled results from two randomised trials. Radiother. Oncol..

[18] Batenburg M.C.T., van der Molen D.R.M., van der Leij F., Doeksen A., van Dalen T., Schoenmaeckers E.J.P., Bijlsma R.M., Witkamp A.J., Ernst M., Sier M.F. (2023). Patient-Reported Symptoms of Late Toxicity in Patients with Breast Cancer Treated with Hypofractionated Radiation Therapy and the Association with Quality of Life. Int. J. Radiat. Oncol. Biol. Phys..

[19] van den Bogaard V.A.B., Spoor D.S., van der Schaaf A., van Dijk L.V., Schuit E., Sijtsema N.M., Langendijk J.A., Maduro J.H., Crijns A.P.G. (2021). The Importance of Radiation Dose to the Atherosclerotic Plaque in the Left Anterior Descending Coronary Artery for Radiation-Induced Cardiac Toxicity of Breast Cancer Patients?. Int. J. Radiat. Oncol. Biol. Phys..

[20] Fodor A., Brombin C., Mangili P., Tummineri R., Pasetti M., Zerbetto F., Longobardi B., Sanchez Galvan A., Deantoni C.L., Dell’OCa I. (2022). Toxicity of Hypofractionated Whole Breast Radiotherapy Without Boost and Timescale of Late Skin Responses in a Large Cohort of Early-Stage Breast Cancer Patients. Clin. Breast Cancer.

[21] Ubeira-Gabellini M.G., Mori M., Palazzo G., Cicchetti A., Mangili P., Pavarini M., Rancati T., Fodor A., del Vecchio A., Di Muzio N.G. (2024). Comparing Performances of Predictive Models of Toxicity after Radiotherapy for Breast Cancer Using Different Machine Learning Approaches. Cancers.

[22] Fodor A., Brombin C., Mangili P., Borroni F., Pasetti M., Tummineri R., Zerbetto F., Longobardi B., Perna L., Dell’oCa I. (2021). Impact of molecular subtype on 1325 early-stage breast cancer patients homogeneously treated with hypofractionated radiotherapy without boost: Should the indications for radiotherapy be more personalized?. Breast.

[23] Cicchetti A., Mangili P., Fodor A., Ubeira Gabellini M.G., Chiara A., Deantoni C., Mori M., Pasetti M., Palazzo G., Rancati T. (2024). Skin dose-volume predictors of moderate-severe late side effects after whole breast radiotherapy. Radiother. Oncol..

[24] Vincenzi M.M., Cicchetti A., Castriconi R., Mangili P., Ubeira-Gabellini M.G., Chiara A., Deantoni C., Mori M., Pasetti M., Palazzo G. (2025). Training and temporally validating an NTCP model of acute toxicity after whole breast radiotherapy, including the impact of advanced delivery techniques. Radiother. Oncol..

[25] Gruppo di Coordinamento AIRO Mammella (2019). Best Clinical Practice Nella Radioterapia dei Tumori Della Mammella 2019. https://www.radioterapiaitalia.it/wp-content/uploads/2019/09/Best-Clinical-Practice-nella-radioterapia-dei-tumori-della-mammella-2019.pdf.

[26] Ahmed W., de Graaf M.A., Broersen A., Kitslaar P.H., Oost E., Dijkstra J., Bax J.J., Reiber J.H.C., Scholte A.J. (2015). Automatic detection and quantification of the Agatston coronary artery calcium score on contrast computed tomography angiography. Int. J. Cardiovasc. Imaging.

[27] Agatston A.S., Janowitz W.R., Hildner F.J., Zusmer N.R., Viamonte M., Detrano R. (1990). Quantification of coronary artery calcium using ultrafast computed tomography. J. Am. Coll. Cardiol..

[28] McCollough C.H., Ulzheimer S., Halliburton S.S., Shanneik K., White R.D., Kalender W.A. (2007). Coronary Artery Calcium: A Multi-institutional, Multimanufacturer International Standard for Quantification at Cardiac CT. Radiology.

[29] Gernaat S.A.M., van Velzen S.G.M., Koh V., Emaus M.J., Išgum I., Lessmann N., Moes S., Jacobson A., Tan P.W., Grobbee D.E. (2018). Automatic quantification of calcifications in the coronary arteries and thoracic aorta on radiotherapy planning CT scans of Western and Asian breast cancer patients. Radiother. Oncol..

[30] Neves P.O., Andrade J., Monção H. (2017). Coronary artery calcium score: Current status. Radiol. Bras..

[31] McClelland R.L., Jorgensen N.W., Budoff M., Blaha M.J., Post W.S., Kronmal R.A., Bild D.E., Shea S., Liu K., Watson K.E. (2015). 10-Year Coronary Heart Disease Risk Prediction Using Coronary Artery Calcium and Traditional Risk Factors: Derivation in the MESA (Multi-Ethnic Study of Atherosclerosis) with Validation in the HNR (Heinz Nixdorf Recall) Study and the DHS (Dallas Heart Study). J. Am. Coll. Cardiol..

[32] Aznar M., Nohria A. (2023). Reducing radiation to the heart in breast cancer: Is that all that matters?. Eur. Heart J..

[33] Belardo A., Dimayuga K.B., Perna L., Fodor A., Giannini L., Mangili P., Palazzo G., Pasetti M., Torrisi M., Tummineri R. (2025). Density and volume of cardiac calcifications detected on planning CT predicts cardiotoxicity after hypo-fractionated whole breast Radiotherapy. Radiother. Oncol..

[34] Li X., Sun Z., Du X., Liu H., Hu G., Xie G. (2018). Bootstrap-Based Feature Selection to Balance Model Discrimination and Predictor Significance: A Study of Stroke Prediction in Atrial Fibrillation. AMIA Annu. Symp. Proc..

[35] Geroldinger A., Lusa L., Nold M., Heinze G. (2021). On resampling methods for model assessment in penalized and unpenalized logistic regression. arXiv.

[36] Buonaccorsi J.P., Romeo G., Thoresen M. (2018). Model-Based Bootstrapping When Correcting for Measurement Error with Application to Logistic Regression. Biometrics.

[37] Collins G.S., Reitsma J.B., Altman D.G., Moons K.G.M. (2015). Transparent reporting of a multivariable prediction model for individual prognosis or diagnosis (TRIPOD): The TRIPOD statement. BMJ.

[38] Enserro D.M., Miller A. (2025). Improving the Estimation of Prediction Increment Measures in Logistic and Survival Analysis. Cancers.

[39] Steyerberg E.W. (2019). Clinical Prediction Models: A Practical Approach to Development, Validation, and Updating.

[40] Arboretti R.G., Salmaso L. (2003). Model Performance Analysis and Model Validation in Logistic Regression. Statistica.

[41] van Velzen S.G.M., Lessmann N., Velthuis B.K., Bank I.E.M., van den Bongard D.H.J.G., Leiner T., de Jong P.A., Veldhuis W.B., Correa A., Terry J.G. (2020). Deep Learning for Automatic Calcium Scoring in CT: Validation Using Multiple Cardiac CT and Chest CT Protocols. Radiology.

[42] Jung W., Shim S.S., Kim K. (2021). CT findings of acute radiation-induced pneumonitis in breast cancer. Br. J. Radiol..

[43] Rancati T., Wennberg B., Lind P., Svane G., Gagliardi G. (2007). Early clinical and radiological pulmonary complications following breast cancer radiation therapy: NTCP fit with four different models. Radiother. Oncol..

[44] Hosseini M., Almasi-Hashiani A., Sepidarkish M., Maroufizadeh S. (2019). Global prevalence of asthma-COPD overlap (ACO) in the general population: A systematic review and meta-analysis. Respir. Res..

[45] Aliberti S., Sotgiu G., Lapi F., Gramegna A., Cricelli C., Blasi F. (2020). Prevalence and incidence of bronchiectasis in Italy. BMC Pulm. Med..

[46] Raghu G., Chen S.-Y., Yeh W.-S., Maroni B., Li Q., Lee Y.-C., Collard H.R. (2014). Idiopathic pulmonary fibrosis in US Medicare beneficiaries aged 65 years and older: Incidence, prevalence, and survival, 2001–11. Lancet Respir. Med..

[47] Choi Y.J., Kim M.J., Lee Y.J., Choi M., Shim W.S., Park M., Kim Y.-C., Kang K.W. (2025). Prevention of radiotherapy-induced pro-tumorigenic microenvironment by SFK inhibitors. Theranostics.

[48] Lind P.A., Bylund H., Wennberg B., Svensson C., Svane G. (2000). Abnormalities on chest radiographs following radiation therapy for breast cancer. Eur. Radiol..

[49] Lind P.A., Wennberg B., Gagliardi G., Fornander T. (2001). Pulmonary complications following different radiotherapy techniques for breast cancer, and the association to irradiated lung volume and dose. Breast Cancer Res. Treat..

[50] Strazzanti A., Trovato C., Gangi S., Marletta D., Milazzotto R., Spatola C. (2019). A Single Institution Study Experience of Secondary Breast Angiosarcoma after Breast Conserving Treatment: Multidisciplinary Management. Int. J. Cancer Clin. Res..

[51] Mascalchi M., Camiciottoli G., Diciotti S. (2017). Lung densitometry: Why, how and when. J. Thorac. Dis..

[52] Lucidarme O., Grenier P.A., Cadi M., Mourey-Gerosa I., Benali K., Cluzel P. (2000). Evaluation of Air Trapping at CT: Comparison of Continuous- versus Suspended-Expiration CT Techniques. Radiology.

[53] Hersh C.P., Washko G.R., Estépar R.S.J., Lutz S., Friedman P.J., Han M.K., Hokanson J.E., Judy P.F., Lynch D.A., Make B.J. (2013). Paired inspiratory-expiratory chest CT scans to assess for small airways disease in COPD. Respir. Res..

[54] Risk Assessment Tools for Severe Side Effects After Breast Radiotherapy: Radiation Safety Through Biological Extended Models and Digital Twins. https://cordis.europa.eu/project/id/101166699/.

