# Supplementary material for: Association of Cardiac and Pulmonary CT Imaging Features with Respiratory Side Effects After Whole-Breast Radiotherapy
Source: Cancers (Basel). 2026 May 25;18(11):1727. doi: 10.3390/cancers18111727 (PMC13255829; doi:10.3390/cancers18111727)
Supplement: Supplementary file 1 [file cancers-18-01727-s001.zip › cancers-4321707-supplementary.pdf]

Supplementary Materials

S1 Complete logistic regression analysis

We present in this section the results of the univariable logistic regression analyses carried out in this study, including dosimetric and densitometric variables derived from pulmonary tissues and the cardiac Max_HU_Heart and Agatston_score. The results are reported separately for the ipsilateral, contralateral and combined lungs, respectively in Table S1, Table S2 and Table S3. In Table S4 is reported the complete univariable analysis of all the VxGy variables, both expressed in cc and percentage with respect to the total ipsilateral volume.

Table S1 Results of the logistic regression carried out on the ipsilateral lung variables.

**Ipsilateral lung**				
	**p-value**	**OR**	**CI95%**	**AUC**
**Volume_Lung**	0.0084	1.0016	[1.001, 0.002]	0.68
**Max_HU_Heart**	0.004	1.003	[1.0015, 1.005]	0.64
**Agatston_score**	0.001	1.001	[1.0005, 0.002]	0.69
**Mean_HU**	0.018	0.987	[0.976, 0.998]	0.67
**Median_HU**	0.029	0.988	[0.978, 0.999]	0.67
**Max_HU**	0.953	0.999	[0.998, 1.002]	0.52
**V850 (cc)**	0.02	1.002	[1.001, 1.003]	0.67
**p10%_HU**	0.019	0.982	[0.967, 0.997]	0.66
**p25%_HU**	0.037	0.986	[0.972, 0.999]	0.65
**p75%_HU**	0.011	0.991	[0.984, 0.998]	0.67
**p90%_HU**	0.04	0.993	[0.986, 0.999]	0.62

Table S2 Results of the logistic analysis conducted on the contralateral lung.

Contralateral lung				
	**p-value**	**OR**	**CI95%**	**AUC**
**Volume_Lung**	0.0087	1.0016	[1.001, 0.003]	0.68
**Max_HU_Heart**	0.004	1.003	[1.0015, 1.005]	0.64
**Agatston_score**	0.001	1.001	[1.0005, 0.002]	0.69
**Mean_HU**	0.002	0.983	[0.976, 0.994]	0.69
**Median_HU**	0.0017	0.982	[0.978, 0.999]	0.70
**Max_HU**	0.754	0.999	[0.998, 1.002]	0.51
**V850 (cc)**	0.018	1.0017	[1.001, 1.003]	0.67
**p10%_HU**	0.0062	0.979	[0.964, 0.997]	0.67
**p25%_HU**	0.0064	0.982	[0.962, 0.996]	0.68
**p75%_HU**	0.0008	0.981	[0.981, 0.998]	0.71
**p90%_HU**	0.014	0.991	[0.986, 0.999]	0.66

Table S3 Results of the logistic analysis carried out on the combined lung structure.

Combined lung structure				
	**p-value**	**OR**	**CI95%**	**AUC**
**Volume_Lung**	0.0033	1.001	[1.0004, 0.0016]	0.70
**Max_HU_Heart**	0.004	1.003	[1.0015, 1.005]	0.64
**Agatston_score**	0.001	1.001	[1.0005, 0.002]	0.69
**Mean_HU**	0.0053	0.985	[0.976, 0.996]	0.68
**Median_HU**	0.029	0.988	[0.978, 0.999]	0.62
**Max_HU**	0.589	0.999	[0.998, 1.002]	0.56
**V850 (cc)**	0.019	1.002	[1.001, 1.003]	0.67
**p10%_HU**	0.012	0.984	[0.963, 0.997]	0.68

**p25%_HU**	0.029	0.983	[0.972, 0.999]	0.66
**p75%_HU**	0.023	0.991	[0.984, 0.998]	0.67
**p90%_HU**	0.042	0.995	[0.986, 0.999]	0.62

Table S4 Results of the logistic regression carried out on volume fractions VxGy (cc and %) of the ipsilateral lungs.

**VxGy in cc**				**VxGy in %**			
**Variable**	**p-value**	**OR**	**AUC**	**Variable**	**p-value**	**OR**	**AUC**
**V10Gy**	0,119	1,004	0,59	**V10Gy**	0,936	1,004	0,51
**V11Gy**	0,116	1,004	0,59	**V11Gy**	0,913	1,006	0,52
**V12Gy**	0,115	1,004	0,59	**V12Gy**	0,898	1,007	0,52
**V13Gy**	0,114	1,004	0,59	**V13Gy**	0,880	1,008	0,52
**V14Gy**	0,116	1,004	0,59	**V14Gy**	0,869	1,008	0,52
**V15Gy**	0,120	1,005	0,58	**V15Gy**	0,867	1,009	0,52
**V16Gy**	0,140	1,004	0,58	**V16Gy**	0,895	1,007	0,52
**V17Gy**	0,170	1,004	0,58	**V17Gy**	0,960	1,003	0,53
**V18Gy**	0,163	1,004	0,58	**V18Gy**	0,929	1,005	0,53
**V19Gy**	0,152	1,004	0,58	**V19Gy**	0,883	1,008	0,53
**V20Gy**	0,149	1,005	0,58	**V20Gy**	0,865	1,010	0,53
**V21Gy**	0,148	1,005	0,58	**V21Gy**	0,852	1,011	0,53
**V22Gy**	0,144	1,005	0,58	**V22Gy**	0,829	1,013	0,53
**V23Gy**	0,143	1,005	0,58	**V23Gy**	0,813	1,014	0,53
**V24Gy**	0,143	1,005	0,58	**V24Gy**	0,796	1,016	0,53
**V25Gy**	0,143	1,005	0,58	**V25Gy**	0,780	1,017	0,54
**V26Gy**	0,139	1,005	0,58	**V26Gy**	0,755	1,019	0,54
**V27Gy**	0,134	1,005	0,58	**V27Gy**	0,726	1,022	0,54
**V28Gy**	0,130	1,005	0,58	**V28Gy**	0,698	1,024	0,54

**V29Gy**	0,125	1,005	0,58	**V29Gy**	0,667	1,027	0,55
**V30Gy**	0,118	1,005	0,58	**V30Gy**	0,628	1,031	0,55
**V31Gy**	0,111	1,006	0,59	**V31Gy**	0,588	1,035	0,55
**V32Gy**	0,104	1,006	0,59	**V32Gy**	0,546	1,040	0,55
**V33Gy**	0,096	1,006	0,59	**V33Gy**	0,502	1,045	0,56
**V34Gy**	0,087	1,006	0,59	**V34Gy**	0,453	1,052	0,56
**V35Gy**	0,076	1,007	0,60	**V35Gy**	0,387	1,062	0,57
**V36Gy**	0,059	1,007	0,61	**V36Gy**	0,290	1,080	0,58
**V37Gy**	0,050	1,008	0,61	**V37Gy**	0,219	1,097	0,59
**V38Gy**	0,081	1,008	0,61	**V38Gy**	0,248	1,095	0,59
**V39Gy**	0,318	1,005	0,58	**V39Gy**	0,547	1,057	0,56
**V40Gy**	0,753	1,003	0,56	**V40Gy**	1,000	1,003	0,46

S2. Bivariable model with V850>175cc and Agatston score

In this Section we present in Table S5 the results of the bivariable model including the dichotomized V850 (>175cc) and the Agatston score of the heart as a continuous variable.

Table S5 Results of the bivariable model concerning the Volume lung and the Agatston score of the heart.

Variable	Var. p-value	Mod. p-value	OR	CI95%	Coeff. β	HL p-value	O.C. AUC (app. AUC)
V850>175cc<br>Agatston score (cont.)	0.0081<br>0.0081	0.0017	3.78<br>1.0011	[1.41,10.6]<br>[1.001,1.005]	1.33<br>0.001	0.092	0.718<br>(0.723)

The combined model showed moderate discrimination and excellent goodness-of-fit. It exhibits an optimism-corrected AUC of 0.718, with an apparent AUC of 0.723 and an estimated optimism of 0.005. Finally, the calibration analysis shows a slightly worst result compared to the couple V850>175cc and

Max_HU_Heart, with a R^2 =0.77, a slope of about 1.15 and a HL p-value of 0.092. Figure S1 displays the predicted probability curves for the proposed model.

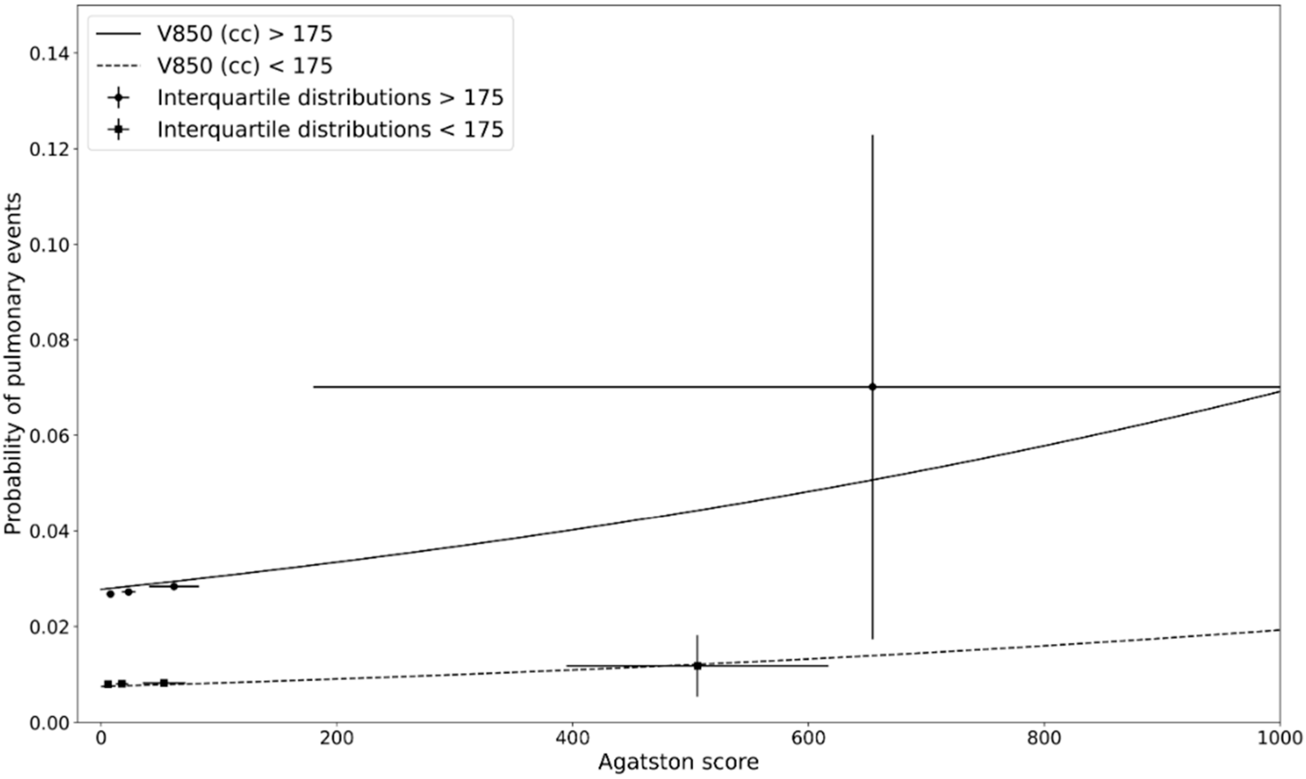

Figure S1 Risk of late pulmonary events: cardiac Agatston score stratified by lung V850 above or below 175cc. The curves represent model-predicted probabilities, while points indicate observed event incidence across cohort quartiles with associated 95%CI.

S3 Calibration plot for the model V850>175cc and Max HU of the heart

In this Section is provided the calibration plot for the bivariable model V850>175cc and the continuous variable Max HU of the heart. Figure S2 illustrates the trend of the line with an associated slope of about 0.95 and a R^2 of 0.847, highlighting an excellent goodness-of-fit.

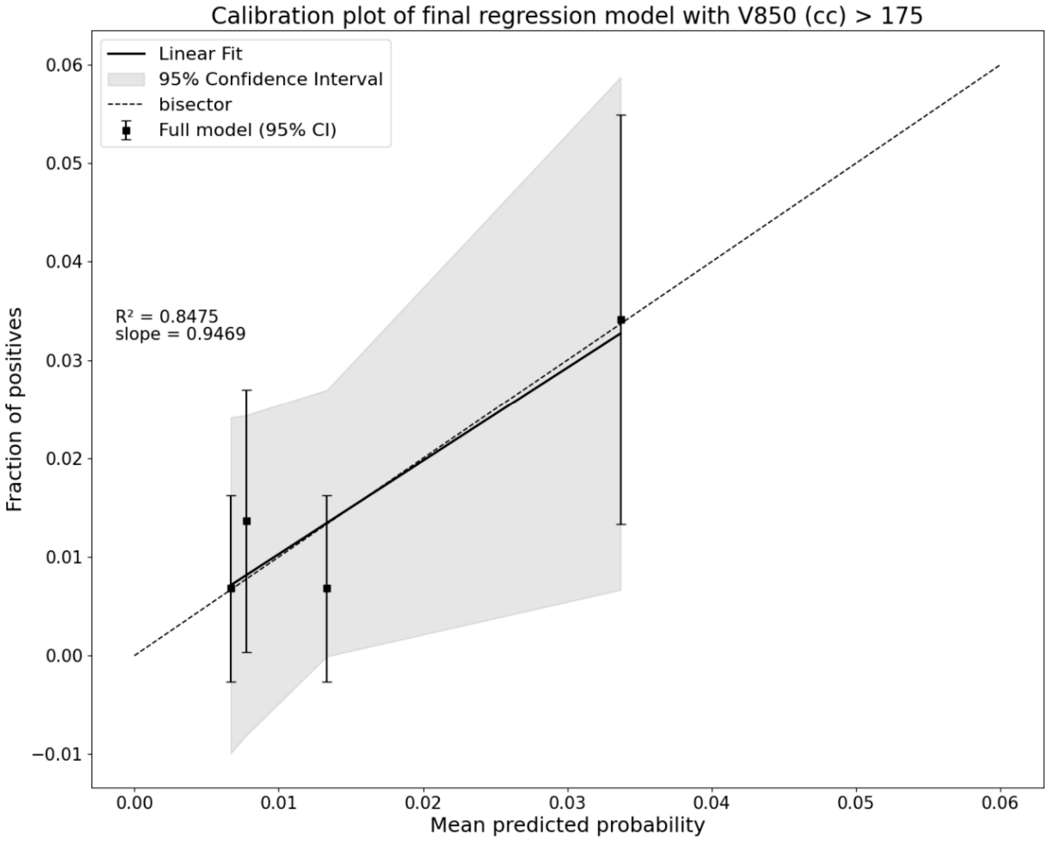

Figure S2 Calibration plot for the selected bivariable model.
